# A Comparison of the Rest Complex Binding Patterns in Embryonic Stem Cells and Epiblast Stem Cells

**DOI:** 10.1371/journal.pone.0095374

**Published:** 2014-04-21

**Authors:** Masahide Seki, Hideki Masaki, Takako Arauchi, Hiromitsu Nakauchi, Sumio Sugano, Yutaka Suzuki

**Affiliations:** 1 Department of Medical Genome Sciences, Graduate School of Frontier Sciences, the University of Tokyo, Kashiwa, Chiba, Japan; 2 Division of Stem Cell Therapy, Center for Stem Cell Biology and Regenerative Medicine, Institute of Medical Science, the University of Tokyo, Minato-ku, Tokyo, Japan; Baylor College of Medicine, United States of America

## Abstract

We detected and characterized the binding sites of the representative Rest complex components Rest, Sin3A, and Lsd1. We compared their binding patterns in mouse embryonic stem (ES) cells and epiblast stem (EpiS) cells. We found few Rest sites unique to the EpiS cells. The ES-unique site features were distinct from those of the common sites, namely, the signal intensities were weaker, and the characteristic gene function categories differed. Our analyses showed that the Rest binding sites do not always overlap with the Sin3A and Lsd1 binding sites. The Sin3A binding pattern differed remarkably between the ES and EpiS cells and was accompanied by significant changes in acetylated-histone patterns in the surrounding regions. A series of transcriptome analyses in the same cell types unexpectedly showed that the putative target gene transcript levels were not dramatically different despite dynamic changes in the Rest complex binding patterns and chromatin statuses, which suggests that Rest is not the sole determinant of repression at its targets. Nevertheless, we identified putative Rest targets with explicitly enhanced transcription upon Rest knock-down in 143 and 60 common and ES-unique Rest target genes, respectively. Among such sites, several genes are involved in ES cell proliferation. In addition, we also found that long, intergenic non-coding RNAs were apparent Rest targets and shared similar features with the protein-coding target genes. Interestingly, such non-coding target genes showed less conservation through evolution than protein-coding targets. As a result of differences in the components and targets of the Rest complex, its functional roles may differ in ES and EpiS cells.

## Introduction

The Rest complex plays a central role in regulating of gene expression through transcription, particularly during early developmental stages. Genetic Rest knock-out generates embryonic lethality on day 11.5 [1]. Rest-mediated transcriptional repression suppresses gene expression for neural genes in non-neural cells, including embryonic stem (ES) cells [1], [2], [3], [4]. The transcription repression is released when neural progenitor cells are committed to enter neural differentiation and thereafter [2], [3]. Recent studies have also elucidated additional diverse functional roles for Rest. In epithelial cancers, lower Rest expression facilitates proliferation and transformation [5]. Conversely, in neural cancers, greater Rest expression facilitates proliferation. In primordial germ cells, Rest represses apoptosis [6]. Indeed, although certain Rest complex functions have been elucidated in detail, the information collected is not always consistent. Singh et al. showed that heterozygous deletion or Rest knock-down induces a pluripotency abnormality in ES cells [7]. In other publications, Rest heterozygous and homozygous knock-out does not have an effect on pluripotency [8], [9]. It also indicated that Rest functions on early differentiation but not maintenance of pluripotency by repressing pluripotent gene expression [10]. Furthermore, the effect of impaired Rest to pluripotency circuitry changes depending on cultivation condition and period after Rest knock-out [11].

In addition to the confusion over Rest function in ES cells, we know even less on the way that Rest gene expression regulation changes during very early development. In this study, we focus on epiblast stem (EpiS) cells derived from the late epiblast of post-implantation embryo [12], [13]. In contrast, it is thought that ES cells correspond to the early epiblast of pre-implantation blastocyst [14]. It is supposed that several distinct molecular mechanisms may explain the characteristic features of ES and EpiS cells. Indeed, ES and EpiS cells have intriguing features. Namely, ES cells require LIF and BMP4 for cell culture, while EpiS cells depend on FGF2 and Activin A [12]. By culturing ES cells under EpiS cell culture conditions, ES cells are converted to EpiS cells [15]. The X chromosome is inactivated in female EpiS cells, but not ES cells. While ES cells contribute to chimera formation, EpiS cells rarely contribute to chimera formation [13], [15]. Importantly, it is supposed that human ES cells are more similar to murine EpiS cells than ES cells. Indeed, studies have shown certain similarities between the signaling pathways for maintenance and epigenetic characteristics in murine EpiS and human ES cells [13], [16], [17]. To understand the pluripotency changes during peri-implantation and regenerative medicine applications, it is essential to investigate the difference between mouse ES and EpiS cells in more detail. Particularly for Rest-mediated regulation, the Rest complex binding patterns in ES and EpiS cells have not been studied, despite their potential importance.

More specifically, the Rest complex functions by modulating chromatin status. While the DNA-binding transcription factor Rest does not have other activities, components of the Rest complex have various histone-modifying enzymatic activities [18]. Namely, the Rest complex includes the histone deacetylase HDAC, which interacts with Rest through Sin3A and CoRest [19], [20]. The H3K4 demethylase Lsd1 and H3K9 methyl transferase G9a also interact through CoRest and Cdyl, respectively [21], [22], [23]. Such histone-modifying enzymes introduce repressive histone modifications to Rest binding sites. Multiple studies have demonstrated that gene repression is not solely mediated through Rest binding, and co-repressor complex binding, such as with Sin3A and CoRest, is essential for such repression. Particularly, Sin3A and Lsd1 are indispensable to early embryonic development. Sin3A and Lsd1 knock-out mice show lethality at days 6.5 and 8.5, respectively [24], [25]. In ES cells, Sin3A knock-down inhibits cellular proliferation [26]. Although Lsd1 knock-out ES cells do not have an obvious phenotype, during differentiation, impaired Lsd1 causes incomplete pluripotency-related gene repression [27]. Studies have also indicated that Rest does not always bind genomic DNA with fixed components. Rather, Rest changes its binding partners occasionally, thereby realizing various functions at its recruitment sites [28].

Herein, we systematically used chromatin-immunoprecipitation with sequencing (ChIP Seq) to better understand the biological meaning of the variations in Rest complex formation and such variations between ES and EpiS cells in mice. We performed ChIP Seq analyses for the Rest complex components Rest, Sin3A, and Lsd1. We also performed the ChIP Seq analyses with representative and active histone modifications. We further examined the expression changes due to Rest binding through transcription start site sequencing (TSS Seq) and mRNA Seq analyses. An integrated analysis of the data shows dynamic Rest complex binding pattern changes during the early developmental stages.

## Materials and Methods

### Cell Culture

E14TG2a ESCs derived from 129/Ola were cultured in GMEM (Sigma-Aldrich) supplemented with non-essential amino acids, 10% Knockout Serum Replacement (KSR; Invitrogen), 1 mM sodium pyruvate, 2 mM L-glutamine, 10^−4^ M 2-mercaptoethanol, 100 U/mL penicillin, 100 µg/mL streptomycin, and 1000 U/mL leukemia inhibitory factor (LIF; ESGRO; Millipore) without feeder cells [29]. The EpiS cells were cultured on murine fibroblast feeders in DMEM/F12 supplemented with 20% KSR, 5 ng/ml bFGF (PeproTech), 10^−4^ M nonessential amino acids, 10^−4^ M β-mercaptoethanol, 2 mM L-glutamax, 100 U/mL penicillin, and 100 µg/mL streptomycin [13].

### Immunohistochemistry

Cultured cells were fixed with 4% paraformaldehyde for 1 hour at room temperature, and permeabilized with PBS containing 0.1% Triton X-100 before blocking with Maxblock blocking medium (Active Motif). Cells were incubated with the following primary antibodies: anti-Nanog (ReproCELL; RCAB001P), anti-Oct4 (SantaCruz; sc-5279), and anti-PECAM1 (BD; 553370). We also used the Alexa Fluor 488 Dye conjugated secondary antibodies (Invitrogen).

### Chromatin Immunoprecipitation and Antibodies

ChIP experiments were performed as described previously [30], [31]. We incubated 1–5×10^7^ cells at room temperature for 10 min with 1% formaldehyde. Cross-linking was terminated by adding 150 mM glycine. The cells were washed twice with PBS and harvested using a cell scraper. For the fixed cells, the cellular membranes were lysed in 5 ml of Lysis Buffer 1 (50 mM HEPES–KOH, pH 7.5, 140 mM sodium chloride, 1 mM EDTA, 10% glycerol, 0.5% NP-40, and 0.25% Triton X-100) with a protease inhibitor cocktail, cOmplete (Roche). The lysates were incubated at 4°C for 10 min and centrifuged at 1,500 rpm for 5 min at 4°C. The nuclear pellets were then resuspended in 5 ml of Lysis Buffer 2 (10 mM Tris–HCl, pH 8.0, 200 mM sodium chloride, 1 mM EDTA, and 0.5 mM EGTA) with a protease inhibitor cocktail, incubated at room temperature for 10 min, and centrifuged at 1,500 rpm for 5 min at 4°C. The nuclear pellets were lysed in 1 ml of Lysis Buffer 3 (10 mM Tris–HCl, pH 8.0, 100 mM sodium chloride, 1 mM EDTA, pH 8.0, 0.5 mM EGTA, pH 8.0, 0.1% sodium deoxycholate, and 0.5% N-lauroylsarcosine) with a protease inhibitor cocktail and sonicated using 18 cycles of 30 s each on ice (TOMY SEIKO; UR-20P). The nuclear lysate was added to 100 µl of 10% Triton-X 100 and centrifuged at 15,000 rpm for 10 min at 4°C.

The supernatant was used as the starting material for the ChIP analyses, and 50 µl of the supernatant was stored as the control (input DNA). Protein A- or G-conjugated magnetic beads (Invitrogen) were washed three times with 1 ml blocking buffer (PBS with 0.5% BSA). The washed beads were resuspended in 250 µl blocking buffer with antibodies and rotated overnight at 4°C. Antibody-conjugated beads were washed three times with 1 ml blocking buffer and resuspended in 100 µl blocking buffer. The suspension was added to the nuclear lysate. The mixtures were rotated overnight at 4°C then washed eight times with wash buffer (50 mM HEPES–KOH, pH 7.5, 500 mM lithium chloride, 1 mM EDTA, 1% NP-40, and 0.7% sodium deoxycholate) and once using TE with 50 mM sodium chloride. The immunoprecipitate eluted with 200 µl of elution buffer (1 M Tris–HCl, pH 8.0, 0.5 M EDTA, pH 8.0, and 1% SDS) at 65°C for 15 min. Two hundred microliters of immunoprecipitate and 50 µl of the input were added to 150 µl elution buffer, which reversed the crosslinking through incubation at 65°C overnight. Two hundred microliters of TE buffer and 8 µl of 10 mg/ml RNase A (Millipore) were added to the samples. The samples were then incubated at 37°C for 2 h. Four microliters of 20 mg/ml proteinase K (Takara) and 7 µl of 300 mM calcium chloride were added to the samples, which were then incubated at 55°C for 2 h. The DNA was isolated through phenol-chloroform extraction and ethanol precipitation. The samples for ChIP Seq using an Illumina HiSeq 2000 were prepared in accordance with the manufacturer’s instructions.

We used the following antibodies: anti-Rest (Millipore; 07–579), anti-Sin3A (Santa Cruz Biotechnology; sc-994), anti-Lsd1 (Abcam; ab17721), anti-RNA Polymerase II (Abcam; ab817), anti-H3K4me3 (Abcam; ab1012), anti-H3ac (Millipore; 06–599), anti-H3K4me1 (Abcam ab8895), anti-H3K27ac (Abcam; ab4729), anti-H3K9me2 (Abcam; ab1220), anti-H3K9me3 (Abcam; ab8898), and anti-H3K27me3 (Millipore; 07–449).

### Small Interfering RNA

The siRNA experiments were performed using Lipofectamine RNAiMAX (Invitrogen) as recommended by the manufacturer. We used the following siRNAs: siRest (Invitrogen; MSS276828) and Stealth RNAi siRNA Negative Control Med GC (Invitrogen). The transfected cells were harvested after 2 days.

### RNA Purification and mRNA Seq

The total RNA was isolated using the RNeasy mini kit and RNase-free DNase set (QIAGEN). The RNA samples for mRNA Seq using an Illumina HiSeq 2000 were prepared in accordance with the manufacturer’s instructions.

### Quantitative PCR and Quantitative Reverse Transcription PCR

Reverse transcription was performed using Superscript II Reverse Transcriptase (Invitrogen) and Oligo dT. The cDNA was quantitated and ChIP-DNA was performed using a 7900HT Fast Real-Time PCR System. The templates were mixed with 2.5 pmol primers and 10 µl Power SYBR Green PCR Master Mix in a 20 µl volume. Using the ΔΔCt method, we calculated the relative expression levels and ChIP recovery efficiency. The primers used herein are shown in Table S1. The primers were designed using Primer3Plus [32]. We also employed primers used in previous studies [33], [34], [35], [36], [37], [38].

### Computational Procedures

The ChIP Seq tags generated were mapped to the reference mouse genome sequences (mm9), and we used the tags that were uniquely mapped, wherein two-base mismatches were allowed. To visualize the ChIP tags, we used the Integrative Genomics Viewer [39], [40]. The transcription factor binding sites were identified using MACS1.4.1 based on the short-read tag information and the ChIP Seq peak detection software [41]. To determine whether consensus sequences are present in the regions surrounding the detected Rest binding sites, we used the motif discovery program MEME [42]. We search known transcription factor binding sites using matrix search program MATCH, matrixes, and vertebrate non redundant minFP cutoffs of TRANSFAC version 2008.3 [43], [44]. The TSS tags were processed as previously described [45]. Briefly, TSS tags in a 500 base bin were clustered and regarded as a single cluster. The tags that belong to each cluster were counted to represent the cluster expression levels. The cluster 50 kb from the 5′-end of a gene model was associated with that model. For the Rest knock-down analysis using mRNA Seq, the mRNA Seq tags were processed using a standard protocol. Briefly, the tags were mapped to the gene regions and counted; we calculated the parts per million tags per kilobase mRNA (rpkm) to measure the expression levels. For the statistical analyses, the representative analytical software “R” was used. The statistical analyses employed to evaluate the significance are indicated in the legends.

### Accession Numbers

The ChIP Seq and mRNA-Seq data herein were deposited in the DDBJ under the following accession numbers: DRA001248 and DRA001249.

## Results and Discussion

### Identifying and Classifying Rest Binding Sites in ES and EpiS Cells

**Figure 1:**
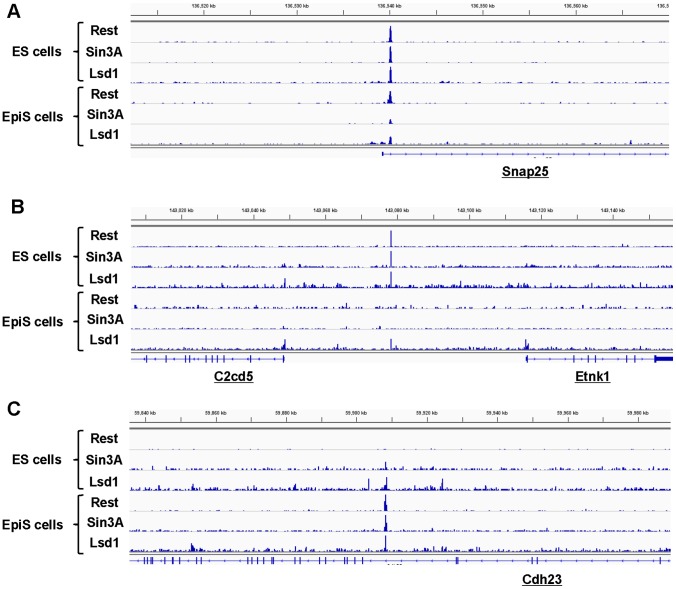
Rest complex ChIP Seq. Examples of the Rest complex binding sites detected and associated with protein-coding genes in ES and EpiS cells. ChIP Seq tags for Rest, Sin3A, and Lsd1 in the indicated cells are shown. (A), (B), and (C) show examples of the ES-unique, common, and EpiS-unique sites in the vicinity of “Snap23”, “C2cd5 and Etnk1”, and “Cdh23”, respectively.

Firstly, we validated re-characterized ES and EpiS cells used in this study. We confirmed the immunohistochemistry and expression patterns of previously reported pluripotency and ES-unique markers [12], [13] (Figure S1 and S2). Using these ES and EpiS cells, to identify genomic localization for the Rest binding sites and representative Rest complex components, namely, Sin3A and Lsd1, in ES cells, we conducted ChIP Seq analyses (for the antibodies used herein and additional details on the experimental conditions, see the Materials and Methods section). We generated at least 10 million ChIP Seq tags for each sample. For peak detection, we used a standard peak detection program, MACS, with the default settings (see Table S2 for the tag statistics). To avoid bias in the tag number between the ChIP and Input data after filtering duplicate tags, we randomly selected tags for which the post-filtering difference was within 10%. The called peaks were further filtered with a p value threshold lower than 10^−10^. We detected 4,632, 7,949, and 13,003 sites for Rest, Sin3A, and Lsd1, respectively (Table 1). When the detected putative Rest binding sites were located within gene regions or 50 kb regions from the RefSeq gene 5′-ends, they were associated with such genes (exemplified in Figure 1). We putatively refer to such sites as “Rest targets” hereafter, although we know that further biological characterization is necessary before such sites can be truly referred to as “targets.”

**Table 1:** Binding sites of Rest complex components.

Coding genes-associated sites	Rest	Sin3A	Lsd1
**ES**	4,632 (5,552)	7,949 (9,371)	13,003 (11,156)
**EpiS**	1,477 (1,823)	3,126 (4,116)	41,093 (16,685)
**Non-coding genes-associated sites**	**Rest**	**Sin3A**	**Lsd1**
**ES**	604 (597)	1,195 (872)	2,156 (1,298)
**EpiS**	165 (165)	372 (359)	5,990 (2,005)

The numbers of binding sites for the indicated factor in ES or EpiS cells detected within the gene regions and 50 kb from the 5′-ends of RefSeq protein-coding gene or RefSeq non-protein-coding genes and putative non-protein-coding genes identified by TSS Seq. The numbers of genes associated with such sites are shown in parentheses.

Consistent with previous studies, we found that the binding sites of the Rest complex components Rest, Sin3A, and Lsd1 did not always overlap. We classified the Rest binding sites into 4 groups depending on the overlapping patterns, namely, 628 Rest-Sin3A-Lsd1 binding sites, 402 Rest-Sin3A binding sites that did not overlap with Lsd1 peaks, 284 Rest-Lsd1 that did not overlap with Sin3A binding sites and 3318 Rest-only binding sites (hereafter, we refer to such sites as R+/S+/L+, R+/S+/L−, R+/S−/L+, and R+/S−/L− sites, respectively; Table 2). We generated a similar dataset for EpiS cells. The statistics for the ChIP Seq tags generated are shown in Table S2. We detected 1,477, 3,126, and 41,093 binding sites for Rest, Sin3A, and Lsd1 (Table 1; exemplified in Figure 1), including 301 R+/S+/L+, 39 R+/S+/L−, 492 R+/S−/L+, and 645 R+/S−/L− sites, respectively (Table 2). In both ES and EpiS cells, the largest population of Rest binding sites was the R+/S−/L− population. To validate that such sites were correctly identified, we conducted independent real-time PCR analyses for four sites in each group of ES and EpiS cells and found that the Sin3A binding intensities for each site were higher in ES than EpiS cells, even at Sin3A binding-undetected sites (Figure S3A–D). Consistent with previous studies, the ChIP Seq signals for the R+/S−/L− sites were typically weaker than for the remaining groups (Figure S3E; see also 26). Previous studies noted that such weak binding sites, which were solely occupied by Rest, may not have biological relevance and lack components with enzymatic activity. Although we also could not interpret the biological meaning of this observation, we removed the R+/S−/L− site both in the ES and EpiS cells from the following analyses.

**Table 2:** Co-binding patterns of Rest complex components.

Coding genes-associated sites	R+/S+/L+	R+/S+/L−	R+/S−/L+	R+/S−/L−
**ES**	628 (997)	402 (676)	284 (472)	3,318 (4,176)
**EpiS**	301 (500)	39 (52)	492 (623)	645 (830)
**Non-coding genes-associated sites**	**R+/S+/L+**	**R+/S+/L**−	**R+/S−/L+**	**R+/S−/L**−
**ES**	76 (80)	69 (77)	44 (55)	415 (451)
**EpiS**	33 (43)	6 (6)	57 (60)	69 (71)

Number of Rest binding sites, associated with coding genes or non-coding genes, categorized by Sin3A and Lsd1 co-binding to the indicated category in ES or EpiS cells. The numbers of genes associated with such sites are shown in parentheses.

### Characterization of the Rest Binding Sites in EpiS and ES Cells

We compared the Rest complex binding patterns detected in ES and EpiS cells (Table 3). Overall, despite using the same parameters to call the “peaks” for both the ES and EpiS cells and that we observed at least the same level of enrichment for the positive controls (Figure S3A), the total number of binding sites and patterns differed remarkably between the cells.

**Table 3:** Overlap of the Rest binding sites between the ES and EpiS cells.

Coding genes-associated sites	Total	R+/S+/L+	R+/S+/L−	R+/S−/L+
**ES-unique**	629 (1,121)	211 (393)	229 (435)	189 (350)
**Common (ES)**	685 (951)	417 (624)	173 (255)	95 (125)
**Common (EpiS)**	773 (1,032)	294 (491)	11 (18)	468 (589)
**EpiS-unique**	59 (82)	7 (10)	28 (34)	24 (38)
**Non-coding genes-associated sites**	**Total**	**R+/S+/L+**	**R+/S+/L**−	**R+/S−/L+**
**ES-unique**	105 (129)	31 (35)	39 (50)	35 (46)
**Common (ES)**	84 (86)	45 (48)	30 (31)	9 (9)
**Common (EpiS)**	85 (104)	31 (35)	4 (4)	50 (54)
**EpiS-unique**	11 (17)	2 (8)	2 (2)	7 (7)

Overlap of the Rest binding sites, associated with cording genes or non-coding genes, between the ES and EpiS cells, except for the R+/S−/L− sites. The numbers of genes associated with such sites are shown in parentheses.

**Figure 2:**
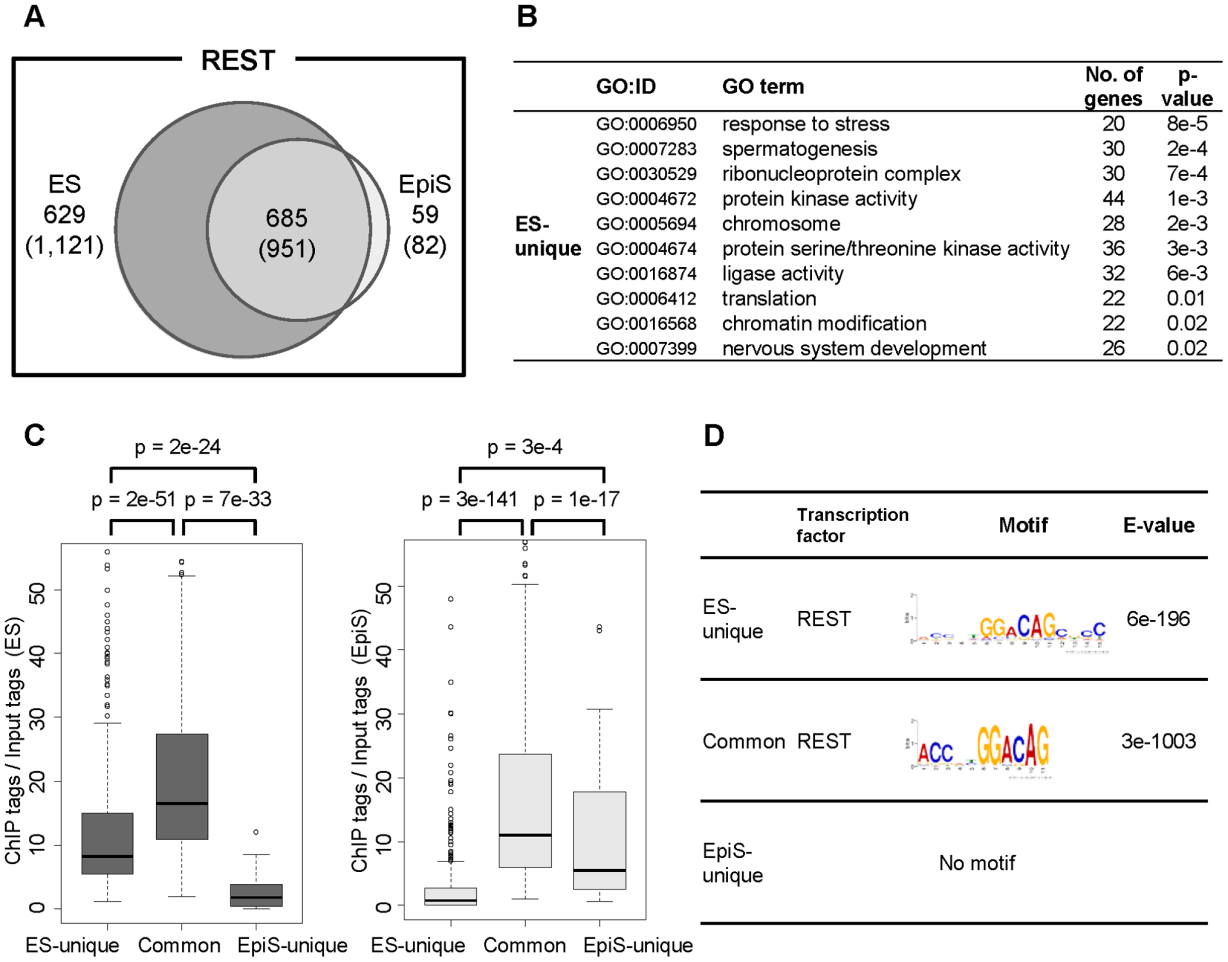
Rest binding site characterization. (A) The Rest binding sites detected through ChIP Seq overlap between the ES and EpiS cells. We used ES cell data as the standard for this comparison. The parentheses show the numbers of protein-coding genes associated with such sites. (B) GO terms enriched for the ES-unique targets. The number of genes in the indicated GO category and statistical significance for such enrichment are shown in the third and fourth columns, respectively. We used GO terms for which the number of genes was 100–500 and the number of Rest target genes in the indicated category was not less than 20. (C) The ES-unique, EpiS-unique, and common detected binding sites intensities using the ChIP tag counts normalized to the input tag counts for the detected binding sites. Boxplots were drawn for the indicated site categories based on the ChIP Seq tag counts in ES (left) and EpiS cells (right). The statistical significance for the differences is also shown in the top margin. (D) The consensus sequences detected around the ES-unique or common Rest binding sites. The sequence logo and statistical significance are shown in the third and fourth columns, respectively.

First, the total number of Rest binding sites was greater in ES cells (1,314 sites) than EpiS cells (832 sites). Approximately half of the Rest binding sites in ES cells were shared with EpiS cells (Figure 2A). However, the unique sites in EpiS cells were rare. Most Rest binding sites in EpiS cells were already bound in the ES cells (685 sites; 92% of the total EpiS-cell binding sites). Rest-mediated regulation may be preferentially used in ES cells and partially used in EpiS cells, but new Rest regulatory mechanisms unique to the EpiS cells were only observed a limited number of instances.

Second, we examined the types of genes included in the ES-unique, common, and EpiS-unique Rest targets (Table S3). As expected, the GO term enrichment analyses showed that neural-developmental-related genes were enriched in the common Rest targets (Table S4). We found that “chromatin modification” or “signal transduction-related” genes are characteristic in ES-unique targets (Figure 2B). The pathway enrichment analysis showed that several neural process-related pathways were enriched in the common Rest targets, while non-neuronal pathways, such as “insulin signaling pathway”, were enriched in the ES-unique targets (Table S5). We also examined and found that Rest is often bound to cell linage marker genes (Table S6). We examined potential Rest binding strength by calculating the ChIP Seq data signal intensities. We found that the binding signals were stronger in the common sites compared with the unique sites in both the ES and EpiS cells (Figure 2C). We further investigated whether consensus sequences were present in the regions that surround the Rest binding sites as detected using the motif discovery program MEME [42]. We detected Rest binding consensus motifs for both the common and ES-unique Rest binding sites (Figure 2D). We did not detect a consensus sequence for the EpiS-unique sites. Furthermore, except for certain differences in the distal consensus sequence regions, we could not detect a clear difference between the ES and EpiS cells, which suggests that factors other than the Rest binding consensus sequence play a role in distinguishing the cells. We further conducted the matrix search of transcription factor binding consensus sequence (TFBS). We detected enrichment of several TFBSs, including that of Oct4, which has been indicated to co-localize with Rest in a previous study [4], were enriched in EpiS-unique sites compared to total Rest sites (Table S7). It may indicate that other transcription factors mediate binding of Rest complex to the genomic DNA.

**Figure 3:**
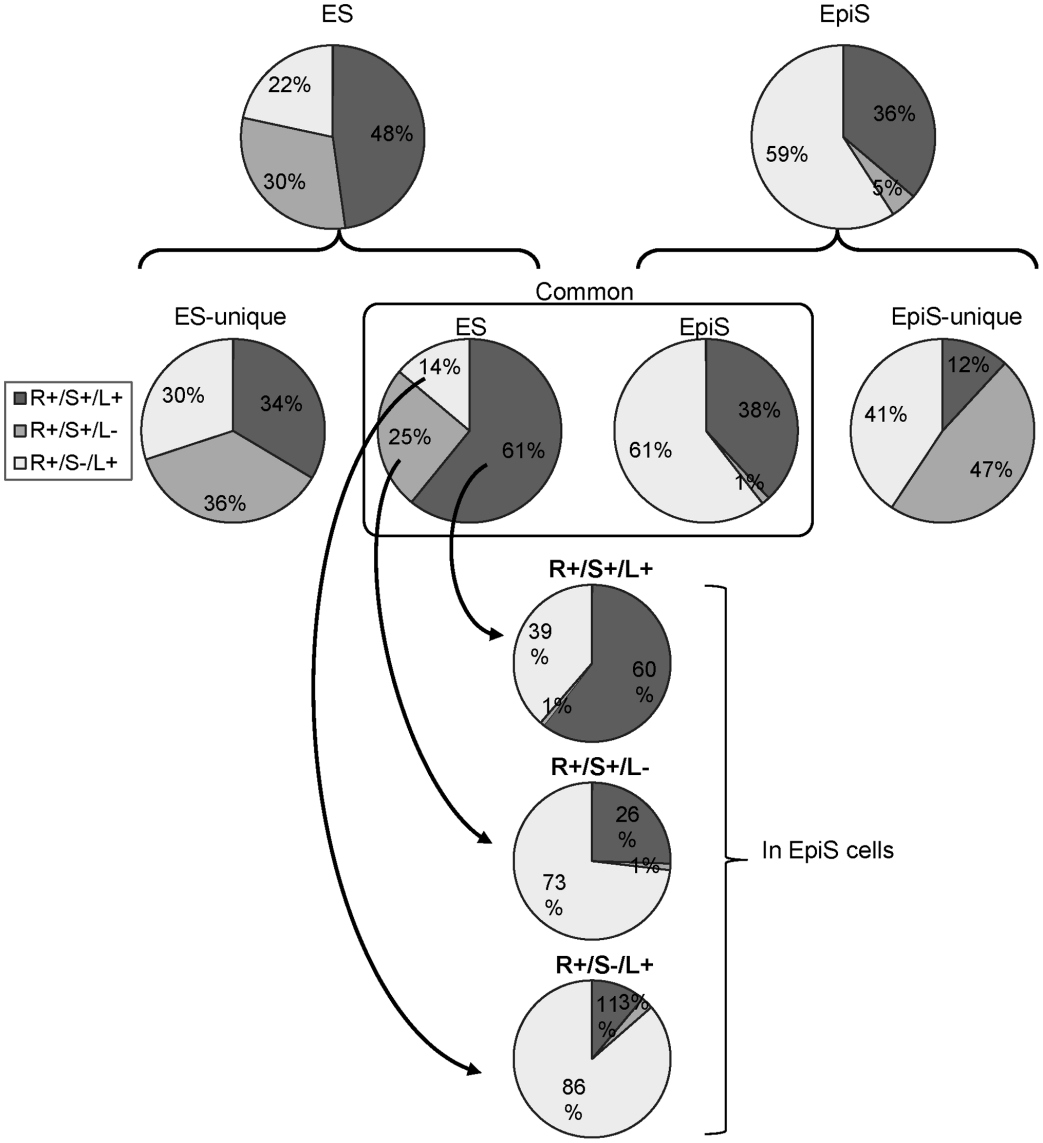
Rest component differences between ES and EpiS cells. The pie chart indicates binding site populations categorized as “R+/S+/L+” (gray), “R+/S+/L−” (pale gray), and “R+/S−/L+” (white). The compositions detected in the ES and EpiS cells are shown at the indicated positions. The top two charts indicate the total Rest binding sites in the ES and EpiS cells. The four lower charts under the braces break down the two top charts into the indicated categories, ES-unique sites, common ES cell sites, common EpiS cell sites, and EpiS-unique sites. The arrow in the common sites indicates the different components between ES and EpiS cells.

**Figure 4:**
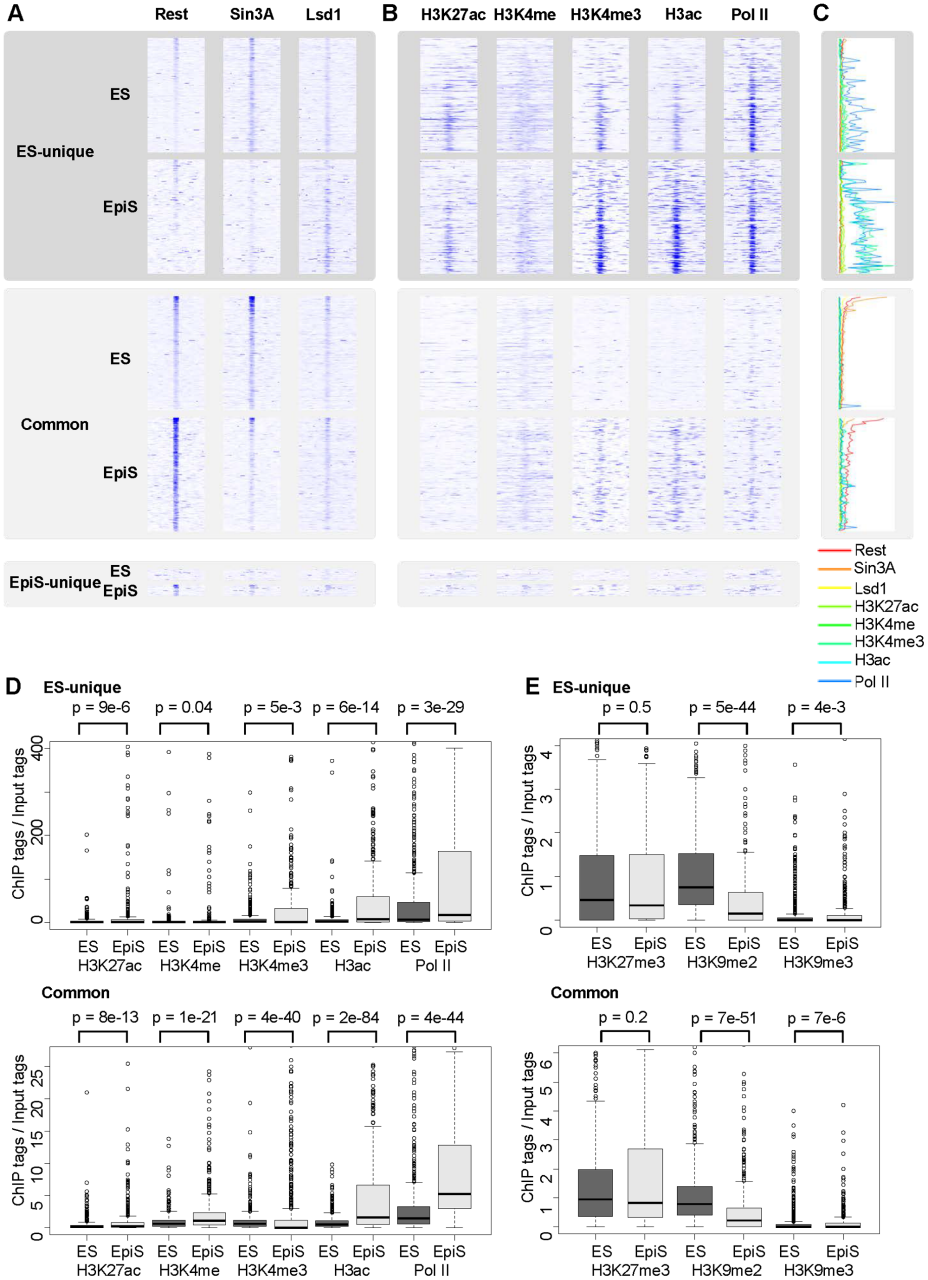
Status of the chromatin in the regions surrounding the Rest binding sites. (A) The ChIP Seq tag count intensities for Rest, Lsd1, and Sin3A in ES or EpiS cells. The ES-unique, EpiS-unique, and common Rest sites are shown in the upper, middle, and lower panels, respectively. Each line represents the −500 base to +500 base region of the detected Rest binding sites. The position where the ChIP tag intensity is 25-fold greater than the input are colored with blue. (B) A heat map of the indicated histone modifications and RNA polymerase II. (C) Vertical section of the ChIP Seq intensities. The color codes for the factors are shown in the bottom margin. (D and E) The active chromatin modification, enhancer modification (D), and repressive modification (E) intensities for the ES and EpiS cells were measured using the ChIP Seq tag counts, which were normalized to the input tag counts. The statistical significance for the differences was evaluated using Wilcoxon’s signed rank test, which is shown in the top margin.

Third, we examined the changes in patterns between ES and EpiS cells for the other complex components, Sin3A and Lsd1 (Figure 3). Our analyses showed that the binding patterns also differed in the ES and EpiS cells. Consistent with the Rest binding sites, the ChIP Seq signal intensities for both Sin3A and Lsd1 were stronger for the common Rest binding sites (Figure 4A). More notably, in ES cells, the R+/S+/L+, R+/S+/L−, and R+/S−/L+ site populations were 628 (48%), 402 (30%), and 284 (22%). The population compositions were 301 (36%), 39 (5%), and 492 (59%) in EpiS cells, respectively (Figure 3; Table 2). Between the cell lines, the R+/S+/L− site differences were most remarkable. While 30% of the Rest binding sites were R+/S+/L− sites, they were only observed at 5% in EpiS cells. We further examined the ES cells pattern changes in more detail among the different populations, namely, the R+/S+/L+, R+/S+/L−, and R+/S−/L+ sites. We observed more R+/S−/L+ sites in each group for the EpiS cells, which almost fully replaced R+/S+/L− and a portion of the R+/S+/L+ sites (Figure 3). Such results suggest that the contribution by Sin3A, which is an HDAC adaptor component, to Rest complex-mediated regulation significantly differs between the ES and EpiS cells.

### The Characteristic Status of the Chromatin that Surrounds the Rest Binding Sites

To examine the status of the chromatin that surrounds the Rest binding sites in ES and EpiS cells, we performed ChIP Seq analyses for the eight types of histone modifications and RNA polymerase II. We generated at least 10 million tags for each sample. The qPCR validation analysis using select cases is shown (Figure S4).

We analyzed the chromatin signature patterns in the regions surrounding the Rest binding sites (Figure 4). For the ES-unique binding sites, we detected significant signals for active chromatin modification, namely, H3K4me3, H3ac, and pol II binding, which were present in ES cells despite clear binding with Rest (Figure 4B, C, and D). Among the different ES cell sites, the signals increased with decreasing Rest-binding ChIP Seq signals. Furthermore, with the transition from ES to EpiS cells, the active chromatin modification signal intensities became more significant with decreasing Rest signals. We also analyzed the repressive modifications (Figure 4E). Consistent with the active chromatin modifications, we detected decreased signals for H3K9me2. Collectively, these results suggest that originally weak Rest binding facilitates transcription at ES-unique sites to a certain extent, and such transcription increases when Rest is not bound in EpiS cells.

For the common sites, Rest binding was detected at similar levels both in ES and EpiS cells. However, the status of the chromatin in the surrounding regions differed between the cells. In the ES cells, we detected virtually no signals for active chromatin modifications. Active chromatin modifications were clearly observed in the EpiS cells. We detected the most significant alterations for H3ac in particular (p<2e-84; Figure 4D). The average H3ac signal intensities changed by 3-fold from ES to EpiS cells. We also detected increased signals for H3K4me3 and pol II (Figure 4D) and decreased signal for H3K9me2 (Figure 4E). For Sin3A depletion, these results suggest that the Sin3A binding levels were remarkably lower from ES to EpiS cells, which yielded lower HDAC activity at the binding sites and, thus, higher H3ac levels, thereby facilitating active chromatin modifications.

In both cell lines, we detected only faint signals of H3K4me for either ES-unique or common binding sites (Figure 4B). Effective enhancers may have been located outside the regions considered herein (50 kb from the RefSeq gene 5′-ends) and not included in our analyses. However, Lsd1, which is an H3K4 demethylase, may remove the H3K4me methyl group in both the ES and EpiS cells. The Lsd1-containing Rest binding site population was high in both the ES and EpiS cells. Furthermore, the Lsd1 binding signals increased from the ES to EpiS cells. Lsd1 may play a role in retaining the transcription-repressed status, which would be fully activated otherwise.

### Changes in the Rest Target Gene Transcription Levels

To examine how the Rest complex formation affects the Rest target transcription levels, we analyzed TSS tag data generated using our unique TSS Seq method. For the TSS Seq, the mRNA cap structure is replaced with Illumina HiSeq sequencing synthetic adaptors to sequence the mRNA immediately downstream of the TSSs [45]. We generated 12 million and 34 million TSS tags from the ES and EpiS cells, respectively. To validate the TSS Seq data, we compared the relative expressions for 13 genes with various expression levels in ES and EpiS cells as measured using RT-qPCR (Figure S2). The TSS Seq and RT-qPCR analysis results were consistent in essentially almost all cases and confirmed an increase or decrease in expression levels from the ES to EpiS cells.

**Figure 5:**
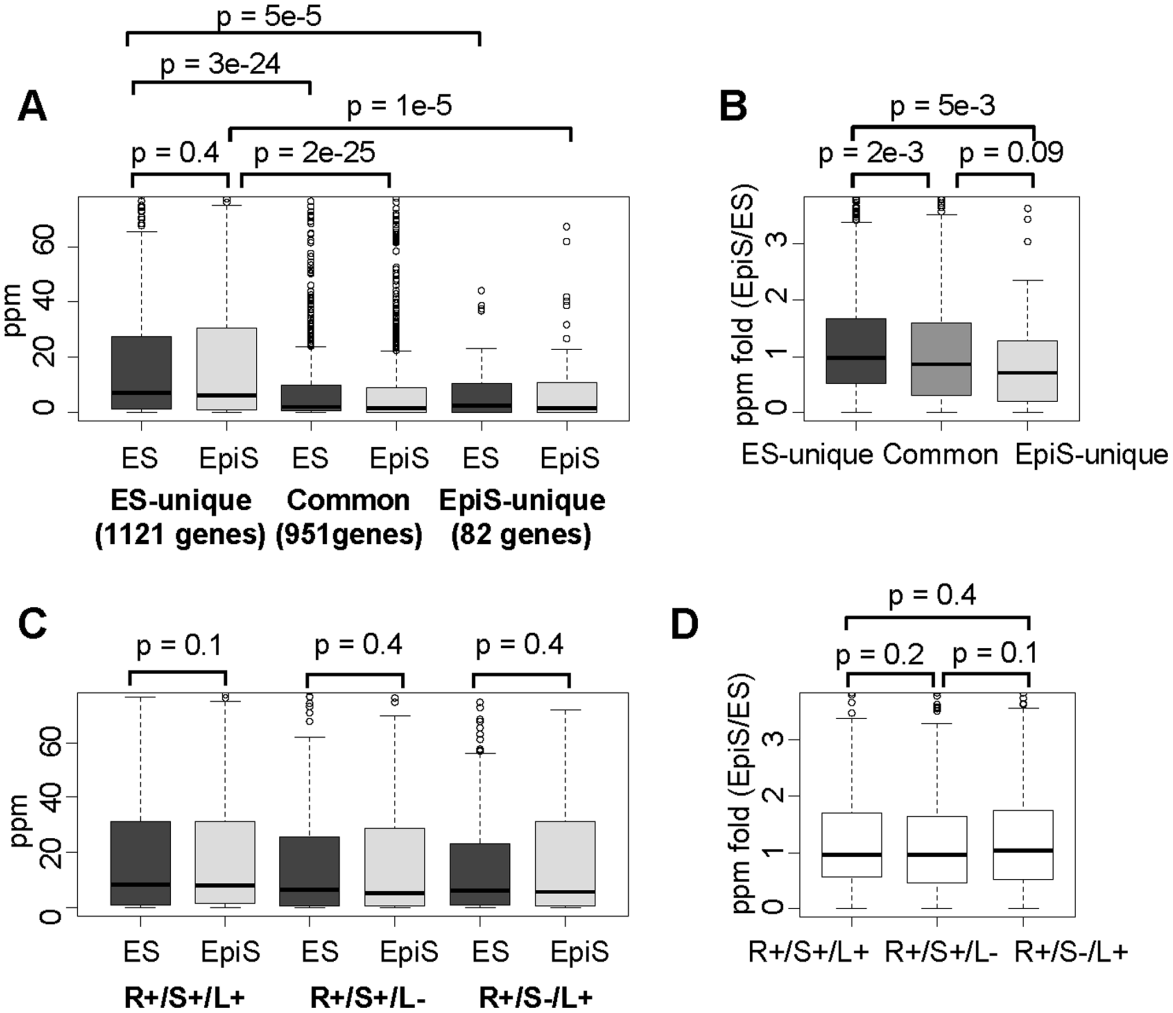
Transcription effects from Rest complex binding. (A and C) Boxplots of the transcript levels measured using TSS tag counts in ES (left lanes) or EpiS cells (right lanes) for the indicated category are shown. (B and D) The TSS tag count fold changes between the ES and EpiS cells for the indicated category are shown. The statistical significance for the differences was evaluated using Wilcoxon’s signed rank test, which is shown in the top margin.

Based on the TSS Seq tag counts, we analyzed the transcript level fold changes from the ES to EpiS cells. We found that the transcripts levels typically increased in the ES-unique targets, which is likely due to a loss of Rest binding in EpiS cells. Although the gene expression level changes were not as high as expected, they were significantly greater than the common and EpiS-unique sites (Figure 5A and B). We also compared the original target genes transcript levels for the ES-unique and common sites in ES cells. We detected higher transcript levels for the ES-unique targets (p<3e-24; Figure 5A). Compared with the ES-unique targets, the common target transcript levels were 2-fold lower, which may reflect stronger Rest binding in this population. We also analyzed the transcript levels between different Rest binding site groups. We did not find distinct features among the R+/S+/L+, R+/S−/L+, and R+/S+/L− sites (Figure 5C and D). Taken together, irrespective of the divergence in the Rest complex components, the consequent transcript levels and the fold changes between the ES and EpiS cells were unexpectedly similar.

### Identification of the ES-unique Rest Targets with Enhanced Transcription in the Rest Knock-down Cells

**Figure 6:**
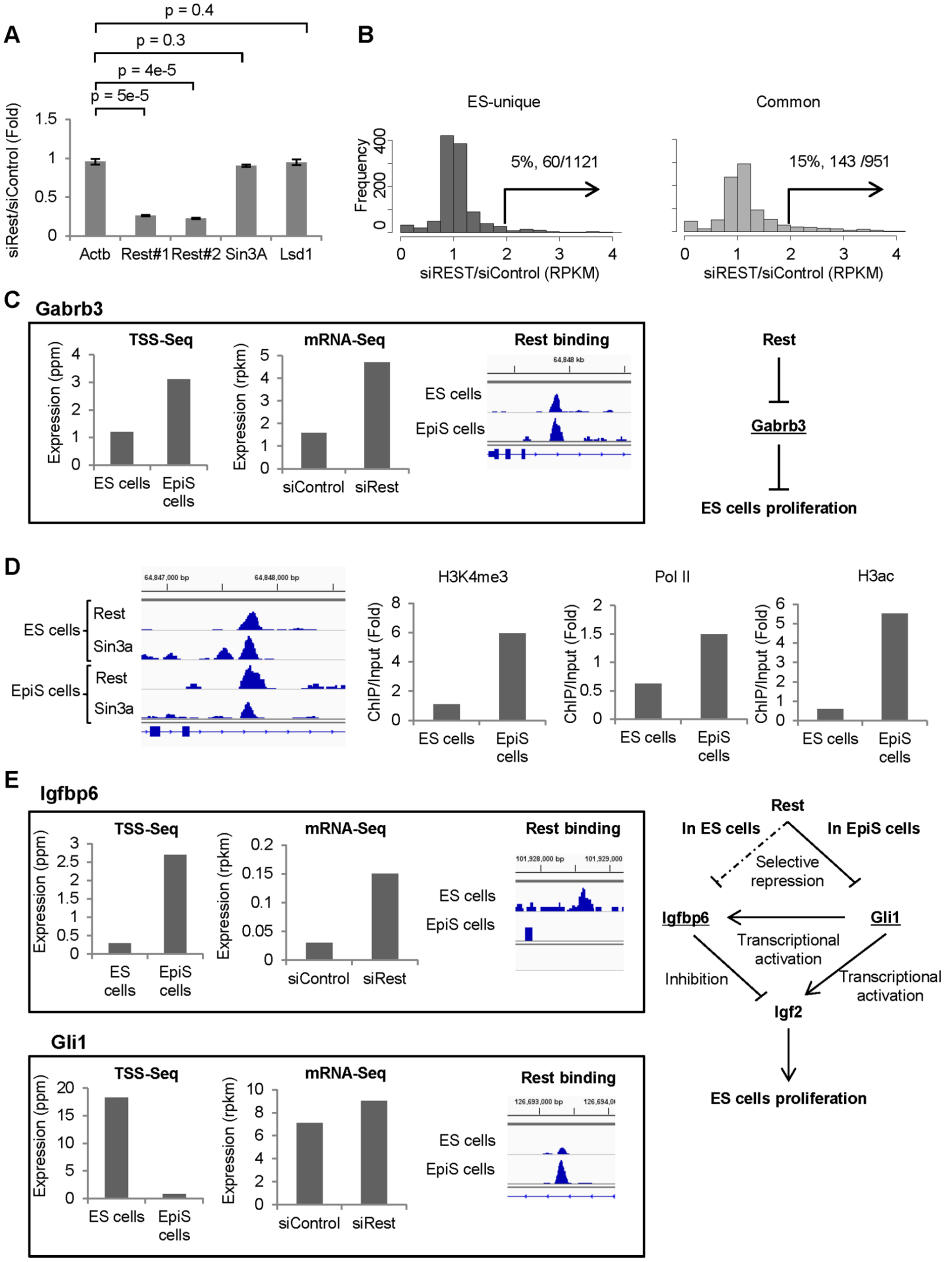
Rest binding sites that affect transcription. (A) Rest knock-down analysis. We show the indicated gene mRNA expression levels measured using RT-qPCR and the Rest results from two independent qPCR primers. (B) The ES-unique (left) and common (right) Rest binding sites distributions that show the indicated fold change in transcription. Populations with greater than a 2-fold increase in transcription are indicated with arrows. (C) The Gabrb3 Rest binding and transcript levels in ES and EpiS cells are compared. Gabrb3 induction through Rest knock-down and the Gabrb3 regulatory network model are also shown. (D) Rest and Sin3A ChIP Seq tags in the indicated cells are shown. Signal intensities of the active chromatin modifications (H3K4me3, RNA Pol2, and H3ac) surrounding Gabrb3 in ES and EpiS cells. (E) The Igfbp6 and Gli1 Rest binding and transcript levels in ES and EpiS cells are compared. The Rest binding sites around Igfbp6 and Gli1 are located in 50 kbp upstream and 80 kbp downstream of each genes, respectively. The Igfbp6 and Gli1 expression change due to Rest knock-down and the Igf2 regulatory network model are also shown.

We focused on the Rest targets with transcript levels that should be repressed primarily by Rest and would be increased without Rest. Thus, we conducted an mRNA Seq analysis using Rest knock-down ES cells (Figure 6A). We compared the mRNA Seq tag count fold changes between the wild type and Rest knock-down ES cells. Indicated in part by the TSS Seq analyses, the enhanced transcription was not always significant in Rest knock-down cells for both ES-unique and common targets (Figure 5A, B, and 6B). These data suggest that Rest-mediated transcription repression may have a buffering effect against decreasing Rest levels. Nevertheless, we selected Rest targets with transcript levels clearly greater than 2-fold enhanced in the Rest knock-down cells. We found 60 (5%) ES-unique and 143 common target genes (15%) for Rest binding (Figure 6B; Table 4). Using RT-qPCR, we confirmed that gene expressions were induced in eight cases (Figure S5). Interestingly, such targets were more frequently selected from common Rest sites than ES-unique sites. Due to enhanced Rest binding at the common sites, such sites may be more susceptible to transcription activation upon Rest knock-down. We further examined the Sin3A and Lsd1 binding patterns at such sites. Such sites were evenly selected from different groups, namely, 19, 11, and 8% common Rest sites and 6, 7, and 3% ES-unique sites from R+/S+/L+, R+/S+/L−, and R+/S−/L+ sites, respectively (Table 4). We also analyzed the chromatin status in the surrounding regions and found no distinct features for the selected sites compared with the total population (data not shown). Such Rest targets, which show enhanced transcription upon Rest knock-down, likely utilize versatile regulatory mechanisms in a similar manner to the additional targets.

**Table 4:** Rest targets with explicit transcription effects.

	Coding genes			Non-conding genes		
Category	R+/S+/L+	R+/S+/L−	R+/S−/L+	R+/S+/L+	R+/S+/L−	R+/S−/L+
**ES-unique (total)**	393	435	350	35	50	46
**ES-unique (induction in REST KO>2)**	23 (6%)	29 (7%)	10 (3%)	0 (0%)	3 (6%)	4 (9%)
**Common (total)**	624	255	125	48	31	9
**Common (induction in REST KO>2)**	117 (19%)	27 (11%)	10 (8%)	7 (15%)	1 (3%)	0 (0%)

The number of ES-unique or common Rest targets in protein-coding and non-protein-coding genes with >2-fold enhanced transcription in Rest knock-down ES cells. The percentages of induced genes from the total genes in each category are described in parentheses.

Among the Rest targets selected, we focused on the targets in the ES cell pluripotency or proliferation pathways. A full list of the identified targets is shown in Table S8. For example, we identified the γ-aminobutyric acid (GABAA) receptor subunit β3 (Gabrb3), which is the major GABAAR subunit in murine ES cells [48]. For this gene, the Rest binding signals were common to both ES and EpiS cells; although, the transcript levels were 2.6-fold greater in ES cells than EpiS cells (Figure 6C). We detected transcript levels greater than 2-fold enhanced in ES cells using the Rest knock-down cells and additional methodologies. We also examined the status of the chromatin surrounding the Rest binding site. We found that active chromatin modifications, including H3ac, were remarkably enhanced in ES cells compared with EpiS cells (Figure 6D). Sin3A binding was weaker in EpiS than ES cells. GABAAR is a differentiation-independent negative proliferation regulator in murine ES cells. Gabrb3 expression was greater in EpiS cells compared with ES cells, which may explain why EpiS cell proliferation is slower than ES cell proliferation. Furthermore, Igfbp6 was an ES-unique target (Figure 6E). Consistently, Igf2 expression in EpiS cells was 20-fold higher than in ES cells. Igfbp6 tightly binds Igf2, thereby inhibiting Igf2 activity [49]. From pathway analysis, we had detected the enrichment of genes belonging to insulin signaling pathways in ES-unique targets (Table S6). This pathway shares many components with the IGF signaling pathway [50]. Rest may also repress the expression of IGF signaling pathway genes downstream of Igf2, in ES cells. Importantly, Igf2 plays an important role in human ES cell self-renewal properties via binding its receptor; it may have same role in mouse EpiS cells with characteristics similar to human ES cells [51]. Interestingly, we also observed that the Rest binding intensity for Gli1 was higher in ES than EpiS cells. Accordingly, the Gli1 transcript levels were 23-fold lower. Gli1 promotes Igf2 transcription [52]. Such opposing signal changes may collectively yield the appropriate level of Igf2 in EpiS cells through feedback regulation.

### Characterization of the Potential Rest Target lncRNAs in EpiS and ES Cells

**Figure 7:**
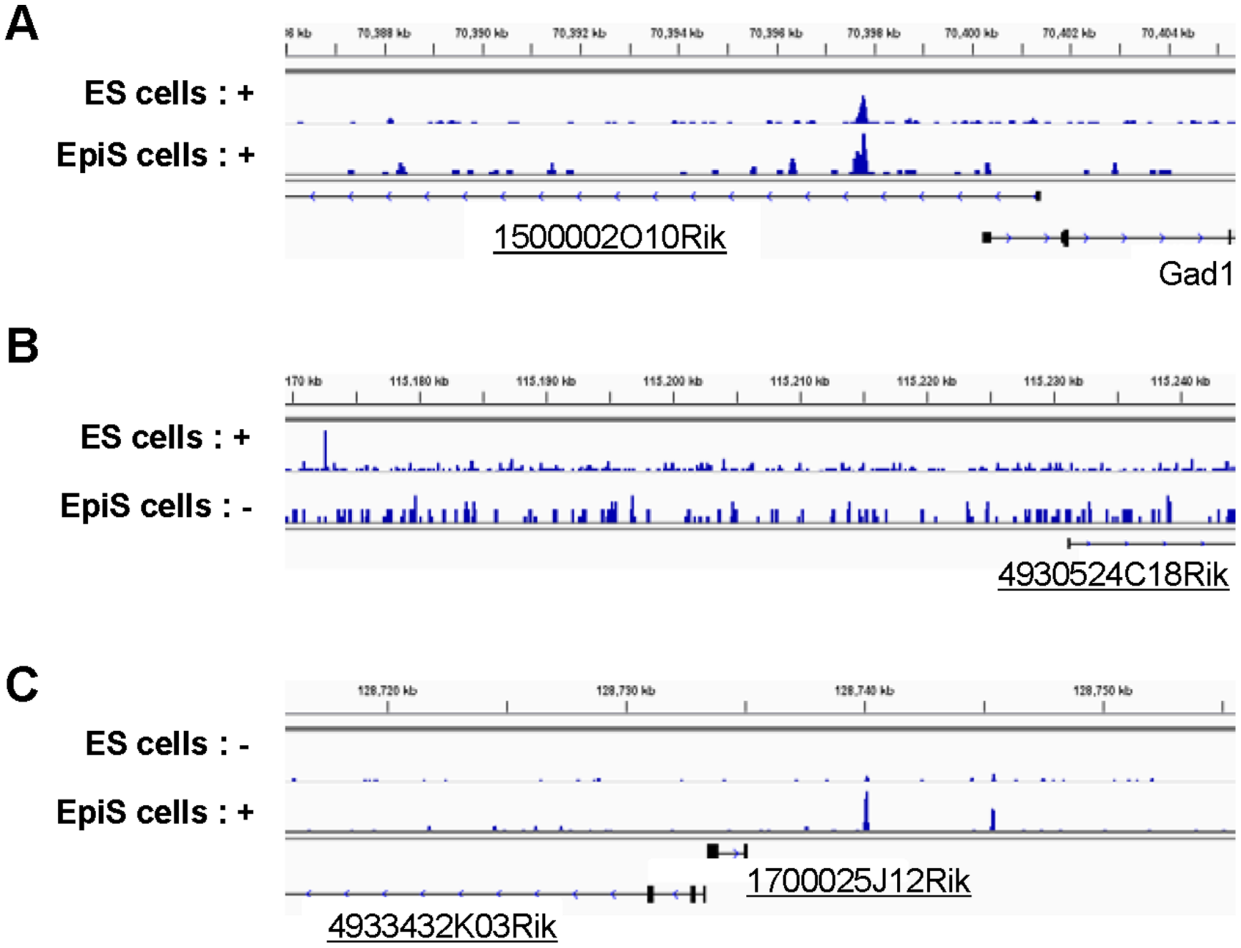
Rest binding sites surrounding the lncRNA. Examples of the Rest binding sites associated with lncRNA in the indicated cell types. Rest ChIP Seq tags in the indicated cells are shown. (A), (B), and (C) show examples of the ES-unique, common, and EpiS-unique site in the vicinity of “1500002O10Rik”, “4930524C18Rik”, and “1700025J12Rik and 4933432K03Rik”, respectively.

**Figure 8:**
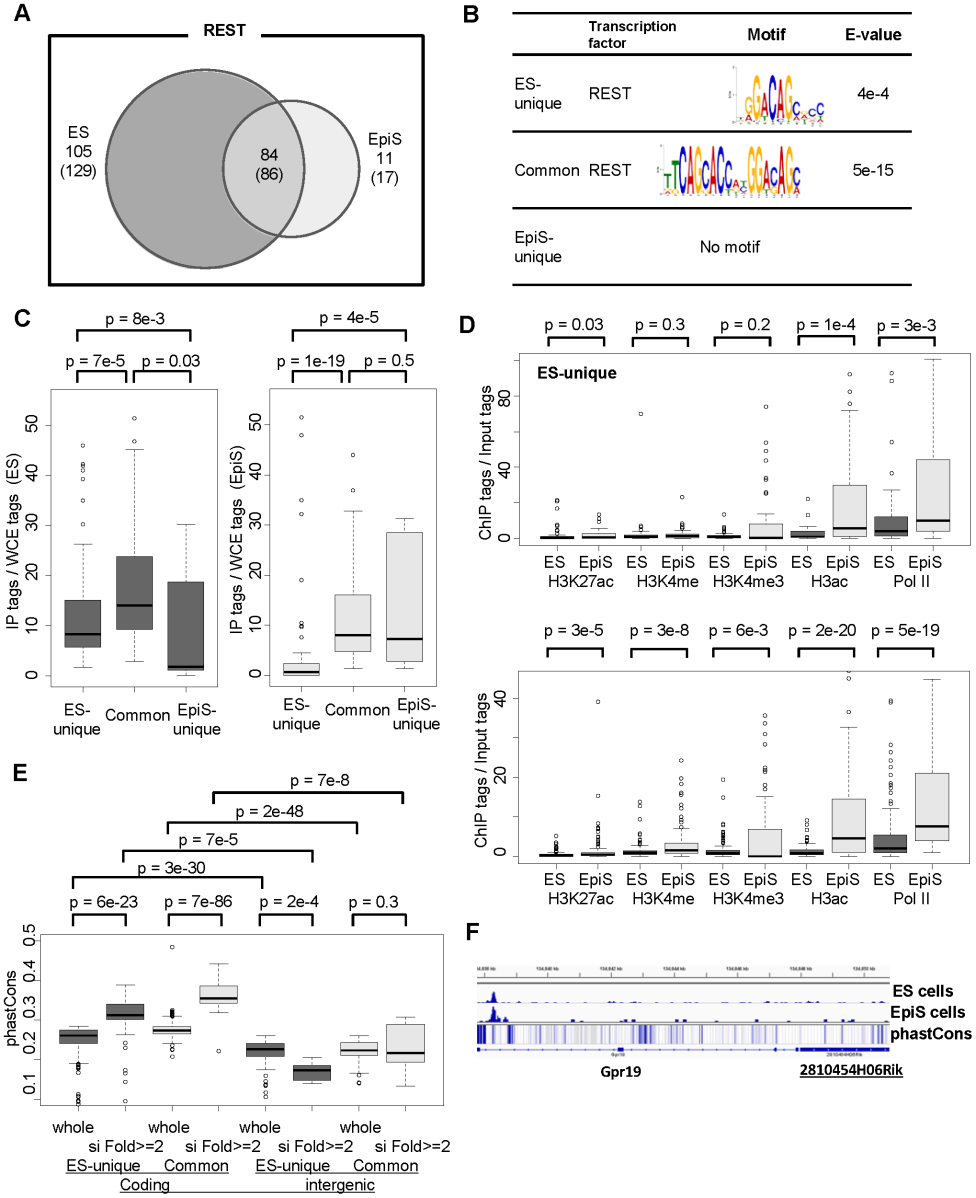
Putative Rest lncRNA targets. (A) Rest binding sites associated with lncRNAs that overlap between ES cells and EpiS cells. We used ES cells data as the standard for this comparison. The numbers of non-coding-genes associated with such sites are shown in parentheses. (B) Consensus sequences surrounding the ES-unique or common Rest binding sites associated with lncRNA. The sequence logo and statistical significance are shown in the third and fourth columns, respectively. (C) Intensities of the detected binding sites measured using the tag counts for the detected binding sites. We show boxplots for the indicated binding site categories based on the ChIP Seq tag counts in the ES (left panel) and EpiS cells (right panel). The statistical significances for the differences are also shown in the top margin. (D) ChIP Seq intensity changes in the active chromatin and enhancer modifications between the ES and EpiS cells. The statistical significance for the differences was evaluated using Wilcoxon’s signed rank test, which is shown in the top margin. (E) Rest binding site evolutionary conservation. We show the average phastCons score distribution from the −50 base to the +50 base in the Rest binding sites [55]. Boxplots are shown for the indicated categories. The statistical significance for the differences was evaluated using Wilcoxon’s signed rank test, which is shown in the top margin. (F) The Rest binding site associated with lncRNA. Rest ChIP Seq tags in the indicated cells are shown in upper two lanes. A heat map of the phastCons score is shown in the third lane, wherein the more conserved sites are bluer.

We also identified and characterized potential Rest targets from intergenic lncRNA. Recent studies showed that lncRNAs are also involved in maintaining ES cell pluripotency in both humans and mice [53], [54]. We associated the Rest binding sites with lincRNAs of RefSeq genes or novel intergenic lncRNAs TSS clusters (TSCs), wherein the TSS tag levels were >5 ppm and not associated with any RefSeq genes (Table 1, 2, and S3; exemplified in Figure 7). The Rest binding patterns surrounding such lncRNAs were similar to the RefSeq TSCs; most targets were unique to the ES cells, and the ChIP Seq signal intensities were greater for the common sites (Figure 8A and C). The Rest binding consensus sequence was only observed in the common and ES-unique sites; the R+/S+/L+, R+/S+/L−, and R+/S−/L+ site sequences varied in a similar manner (Figure 8B). Furthermore, the chromatin status trends in the surrounding regions were similar to the protein-coding genes (Figure 8D). Collectively, such observations suggest that lncRNA transcription may be regulated in a similar manner to the protein-coding genes. A previous report has suggested that Rest directly represses miR21 and consequently contributes maintenance of pluripotency in ES cells [7], but this notion had been challenged in later studies [4], [8]. We also detected Rest binding site around miR21 neither in ES nor EpiS cells (Table S3 and Figure S6). Although mRNA Seq could not detect mature microRNA, we could rarely detect induction of pri-miR21 in Rest knock-down. We show the binding information of Rest for other miRNAs in Table S3.

Similar to the protein-coding genes, we searched for Rest targets with explicit transcription consequences upon Rest knock-down. We found 7 and 8 such cases in the ES-unique and common Rest sites, respectively (Table 3B; Table S8). A full list of the select Rest target lncRNAs is shown in Table S8. Interestingly, we found that the level of evolutionary conservation differed significantly between the different Rest-binding-site groups (Figure 8E). The most significant evolutionary conservation was detected for the protein-coding genes, which showed over 2-fold enhanced transcription in the Rest knock-down cells. The level of evolutionary conservation was lowest for the Rest binding sites associated with lncRNAs. As exemplified in Figure 8F, despite clear Rest-binding signals in the surrounding regions and enhanced transcript levels in the Rest knock-down cells, the genomic regions with lncRNAs transcripts showed low evolutionary conservation. This lack of evolutionary conservation has been reported in humans for certain genes. For example, in humans, the lncRNA HOTAIR plays a well-characterized role in human ES cells as a scaffold for the polycomb repressive complex. However, in mice, the genome sequence is poorly conserved, which suggests an evolutionary loss of this gene [56]. Further experimental validation of such evolutionarily divergent lncRNAs is necessary to better understand lncRNA involvement in Rest-mediated regulation for the respective organisms.

## Conclusion

In this study, we identified and characterized putative binding site patterns for the representative Rest complex components Rest, Sin3A, and Lsd1. We characterized the Rest binding sites in ES and EpiS cells and found the following. 1) The ES-unique and common Rest binding sites regulated different functional gene categories; the Rest binding signal intensities and transcription repression levels were most remarkable for the common Rest binding sites. 2) The co-binding patterns of the pivotal Rest complex components, Sin3A and Lsd1, and status of the chromatin in the surrounding regions were diverse. 3) Among the Rest targets, the genes that exhibited enhanced transcription upon Rest knock-down were unexpectedly low in number; nevertheless, we identified 203 and 15 such Rest targets from protein-coding genes and putative lncRNA genes, respectively. Taken together, our Rest complex data suggest that this complex is involved in transcription regulation through diverse mechanisms and occasional changes in chromatin modifications. Additional extensive functional characterization of the protein-coding and non-protein coding genes identified as putative Rest targets for which transcription is affected should enhance our understanding of the molecular mechanisms underlying the similar but distinct phenotypes in ES and EpiS cells.

## Supporting Information

Figure S1 **Definition and characterization of the ES and EpiS cells.** Immunochemistry of ES (top) and EpiS cells (bottom) for undifferentiated state markers (Nanog and Oct4) and ES specific marker (Pecam1) [13].
(TIFF)

Figure S2 **TSS Seq validation analysis.** A comparison of the transcript level fold changes from ES to EpiS cells between TSS Seq and RT-qPCR. For RT-qPCR, we used the ΔΔCt method with primers designed for the indicated genes and used the Actb gene as the control [35], [37] (Table S1). Rex1, Klf4, and Tbx3 are ES cells specific markers. Eomes, Cer1, and Fgf5 are EpiS cells specific markers.
(TIFF)

Figure S3 **ChIP result validation analysis for the Rest complex components.** (A–C) qPCR validation of Rest complex binding sites categorized as R+/S+/L+ (A), R+/S+/L− (B), R+/S−/L+ (C), and R+/S−/L− (D). We showed the fold enrichment of Rest (dark gray), Sin3A (pale gray), and Lsd1 (gray) at the Rest binding sites associated with the indicated genes. The fold enrichment was calculated using the ΔΔCt method. We used primers designed against binding sites from each category and associated with the indicated genes, and primers were designed against the intergenic regions 1 as a control (Table S1). (E) The Rest binding intensities for “R+/S−/L−” and other types of Rest binding sites. Boxplots are used for the indicated “peak” categories based on the ChIP Seq tag counts in the ES (left) and EpiS cells (right). The statistical significances for the differences are also shown in the top margin.
(TIFF)

Figure S4 **ChIP validation analysis for the histone modifications.** qPCR validation of the RNA polymerase II and histone modifications in ES and EpiS cells. As a positive control for the active promoters (Pol2, H3K4me3, and H3ac) and enhancers (H3K4me1 and H3K27ac), we employed primers that target the H3K4me3 and p300 binding sites and that were used in previous studies [33], [34] (Table S1). As a negative control, we used intergenic region 1 primers, which were also used to validate the Rest complex observations. We used primers designed against the Rps27a promoter as a positive control for Pol2 binding. For H3K9me2, we used primers designed for Rps27a promoter as negative control and Mage-a2 promoter and intergenic region 2 as positive control [36]. For the repressive modifications, H3K9me3 and H3K27me3, we used primers designed for the Hox region as a positive control and the primers referred to as active promoter 1 as a negative control [34].
(TIFF)

Figure S5 **mRNA Seq validation analysis in Rest knockdown cells.** The fold change in Rest target gene transcript levels using Rest knock-down and measured using mRNA Seq (dark gray) and qRT-PCR (pale gray) in ES cells. For RT-qPCR, we used the ΔΔCt method with primers designed for the indicated genes and used the Gapdh gene as the control [38] (Table S1).
(TIFF)

Figure S6 **Validation of the effect of Rest to miR21.** (A) Signal intensities of Rest binding on Rest binding site around miR21, indicated on [7], in ES and EpiS cells. (B) miR21 induction through Rest knock-down is shown.
(TIFF)

Table S1 **Primers used in this study.** The primer sequences for ChIP-qPCR and RT-qPCR are shown.
(XLSX)

Table S2 **A summary of the ChIP-Seq tag and peaks.** (A) A summary of the number of tags and peaks called using ChIP Seq MACS for the Rest complex components Rest, Sin3A, and Lsd1 in ES and EpiS cells. The tag numbers from sequencing used for peak detection are shown in the second and third columns. The MACS-called peaks filtered using a p value threshold lower than 10^−10^ are shown in the fourth and fifth columns. (B) A summary of the number of tags from ChIP Seq for RNA polymerase II and eight types of histone modifications in ES and EpiS cells.
(TIFF)

Table S3 **A list of Rest binding genes.** Each sheet includes a list of the ES-unique, EpiS-unique, and common Rest binding genes for protein-coding and non-protein-coding genes. The internal IDs and RefSeq information are shown in the first and second through fourth columns. The transcript levels measured using mRNA Seq in control and Rest knockdown ES cells are shown in the fifth and sixth column. The transcript levels measured using TSS Seq in ES and EpiS cells are shown in the seventh and eighth column. The transcript position and sequences are shown in the ninth through twelfth columns.
(XLSX)

Table S4 **A GO enrichment analysis of common sites.** The GO terms enriched for the common sites. The number of genes in an indicated GO category and the statistical significance for such enrichment are shown in the third and fourth columns, respectively. We used GO terms for which the number of genes was 100–500 and the number of Rest target genes in the indicated category was not less than 20.
(TIFF)

Table S5 **Pathway analysis of Rest binding sites.** The KEGG pathways enriched for the common sites (A) and ES-unique sites (B) [46]. The number of genes in an indicated pathway and the statistical significance for such enrichment are shown in the third and fourth columns, respectively.
(TIFF)

Table S6 **Linage marker genes associated with Rest binding sites.** A list of Rest binding genes among linage specifically up-regulated genes described in [47]. Cell linages, where indicated marker genes are up-regulated, are shown in the first column. The internal IDs and RefSeq information are shown in the second and third through fifth columns. The fold increase upon Rest knock-down is shown in the sixth column. The transcript position and sequences are shown in the seventh through tenth columns. The cell specificity category of Rest binding is shown in the eleventh column.
(XLSX)

Table S7 **Transcription factor binding consensus motif enriched in Epi-unique sites.** The transcription factor binding consensus sequences enriched for the EpiS-unique sites relative to total Rest binding sites. The number of sites harboring consensus sequence of indicated transcription factor and the statistical significance for such enrichment are shown in the third and fourth columns, respectively.
(TIFF)

Table S8 **A list of putative Rest target genes.** Each sheet includes a list of the ES-unique and common Rest target genes for protein-coding and non-protein-coding genes with >2-fold enhanced transcription upon Rest knock-down. The internal IDs and RefSeq information are shown in the first and second through fourth columns. The fold increase upon Rest knock-down is shown in the fifth column. The transcript position and sequences are shown in the sixth through ninth columns. For the non-protein-coding genes, the cell specificity category is shown in the tenth column.
(XLSX)
