# Supplementary figures and images for: A Comparison of the Rest Complex Binding Patterns in Embryonic Stem Cells and Epiblast Stem Cells
Source: PLoS One. 2014 Apr 21;9(4):e95374. doi: 10.1371/journal.pone.0095374 (PMC3994037; doi:10.1371/journal.pone.0095374)

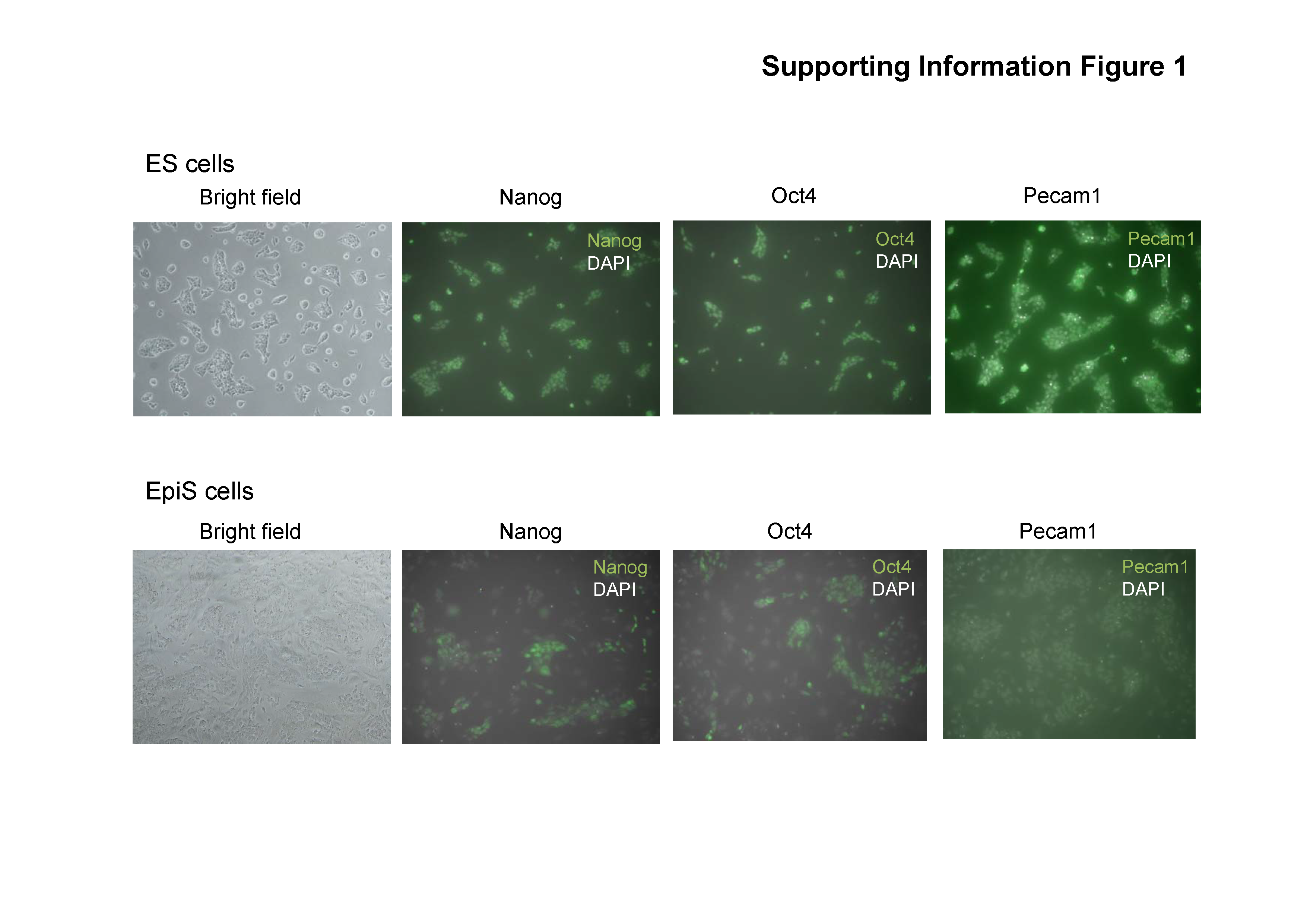

Supplement: Figure S1 — Definition and characterization of the ES and EpiS cells. Immunochemistry of ES (top) and EpiS cells (bottom) for undifferentiated state markers (Nanog and Oct4) and ES specific marker (Pecam1) [13]. (TIFF) [file pone.0095374.s001.tif]

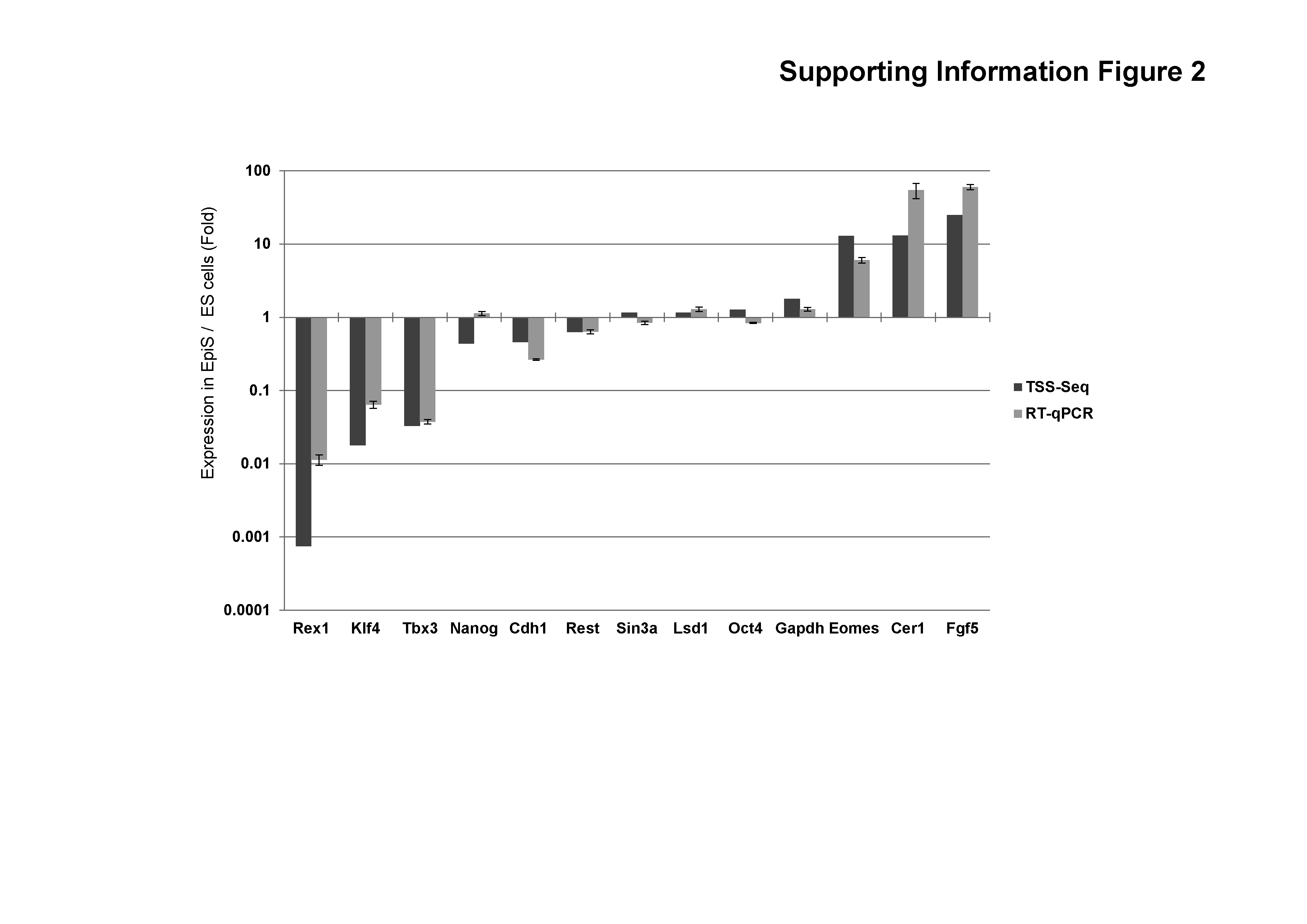

Supplement: Figure S2 — TSS Seq validation analysis. A comparison of the transcript level fold changes from ES to EpiS cells between TSS Seq and RT-qPCR. For RT-qPCR, we used the ΔΔCt method with primers designed for the indicated genes and used the Actb gene as the control [35], [37] (Table S1). Rex1, Klf4, and Tbx3 are ES cells specific markers. Eomes, Cer1, and Fgf5 are EpiS cells specific markers. (TIFF) [file pone.0095374.s002.tif]

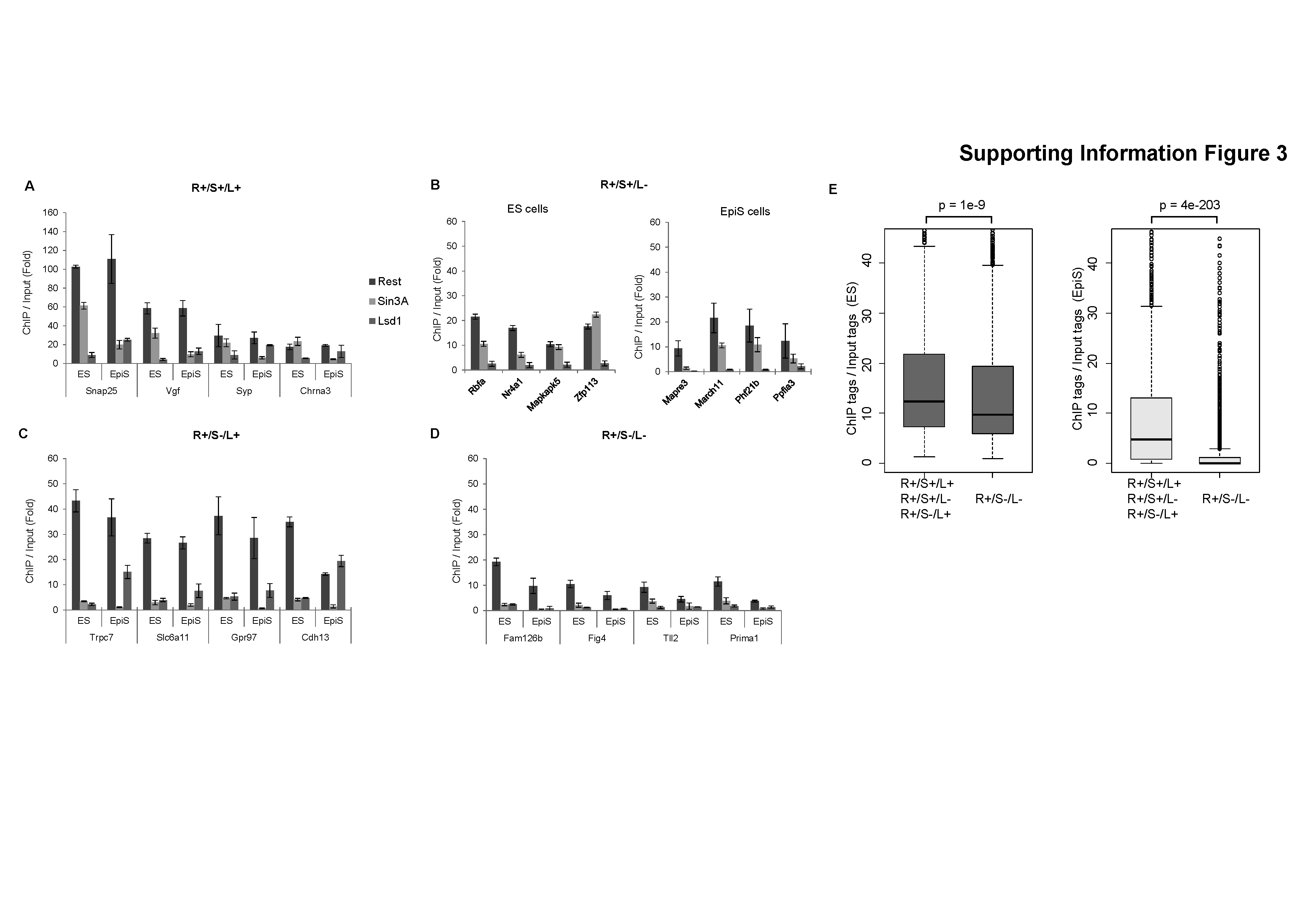

Supplement: Figure S3 — ChIP result validation analysis for the Rest complex components. (A–C) qPCR validation of Rest complex binding sites categorized as R+/S+/L+ (A), R+/S+/L− (B), R+/S−/L+ (C), and R+/S−/L− (D). We showed the fold enrichment of Rest (dark gray), Sin3A (pale gray), and Lsd1 (gray) at the Rest binding sites associated with the indicated genes. The fold enrichment was calculated using the ΔΔCt method. We used primers designed against binding sites from each category and associated with the indicated genes, and primers were designed against the intergenic regions 1 as a control (Table S1). (E) The Rest binding intensities for “R+/S−/L−” and other types of Rest binding sites. Boxplots are used for the indicated “peak” categories based on the ChIP Seq tag counts in the ES (left) and EpiS cells (right). The statistical significances for the differences are also shown in the top margin. (TIFF) [file pone.0095374.s003.tif]

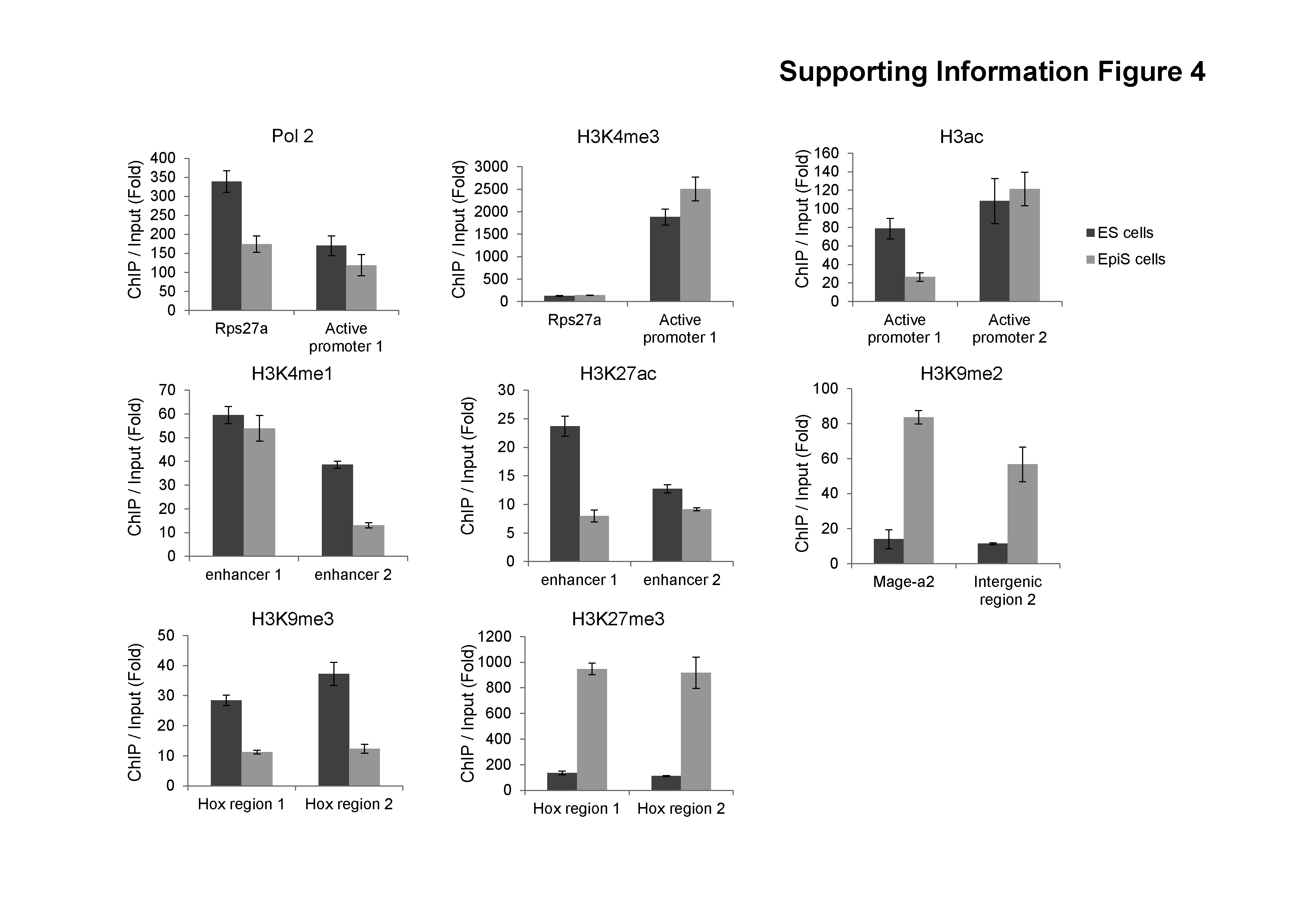

Supplement: Figure S4 — ChIP validation analysis for the histone modifications. qPCR validation of the RNA polymerase II and histone modifications in ES and EpiS cells. As a positive control for the active promoters (Pol2, H3K4me3, and H3ac) and enhancers (H3K4me1 and H3K27ac), we employed primers that target the H3K4me3 and p300 binding sites and that were used in previous studies [33], [34] (Table S1). As a negative control, we used intergenic region 1 primers, which were also used to validate the Rest complex observations. We used primers designed against the Rps27a promoter as a positive control for Pol2 binding. For H3K9me2, we used primers designed for Rps27a promoter as negative control and Mage-a2 promoter and intergenic region 2 as positive control [36]. For the repressive modifications, H3K9me3 and H3K27me3, we used primers designed for the Hox region as a positive control and the primers referred to as active promoter 1 as a negative control [34]. (TIFF) [file pone.0095374.s004.tif]

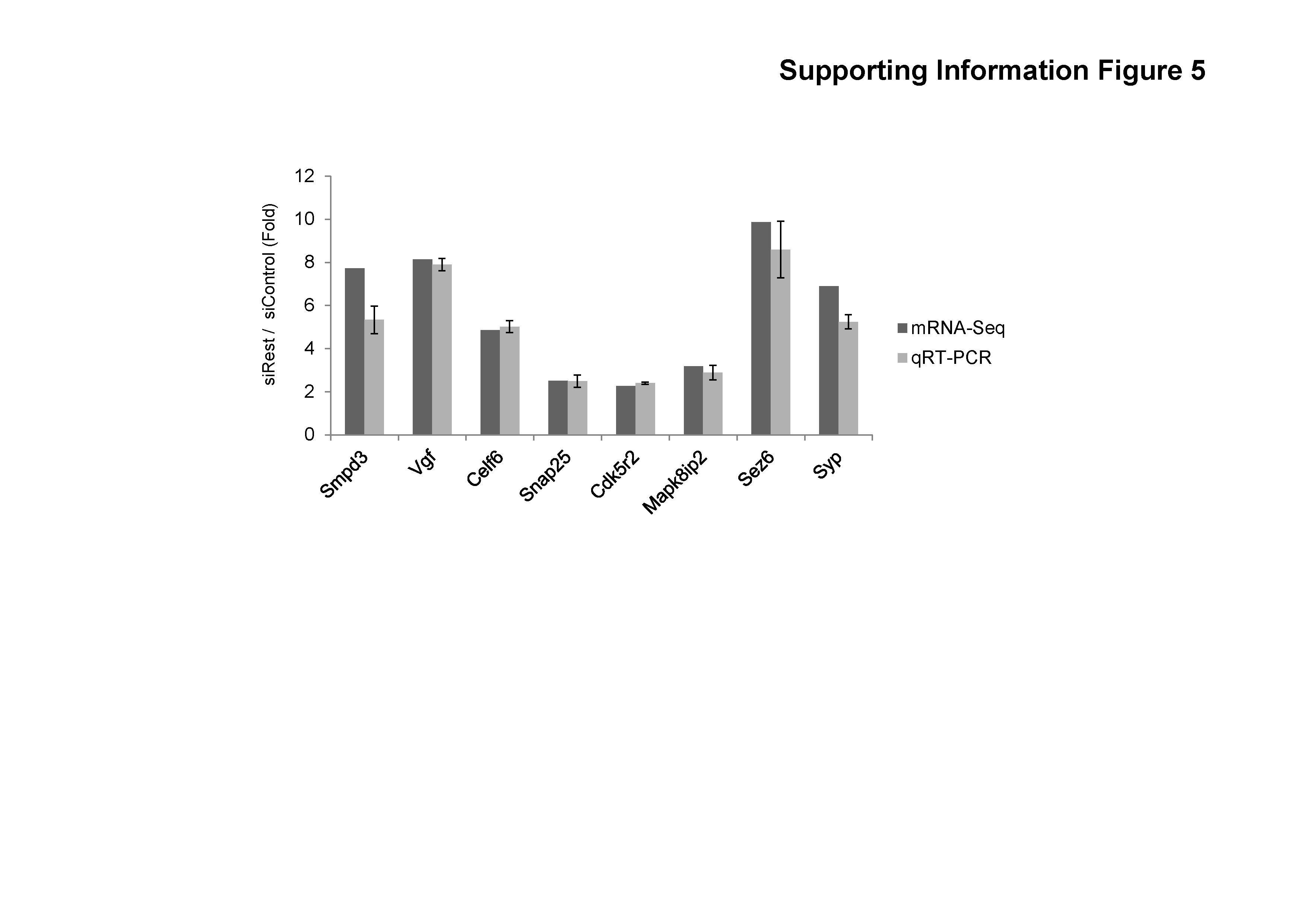

Supplement: Figure S5 — mRNA Seq validation analysis in Rest knockdown cells. The fold change in Rest target gene transcript levels using Rest knock-down and measured using mRNA Seq (dark gray) and qRT-PCR (pale gray) in ES cells. For RT-qPCR, we used the ΔΔCt method with primers designed for the indicated genes and used the Gapdh gene as the control [38] (Table S1). (TIFF) [file pone.0095374.s005.tif]

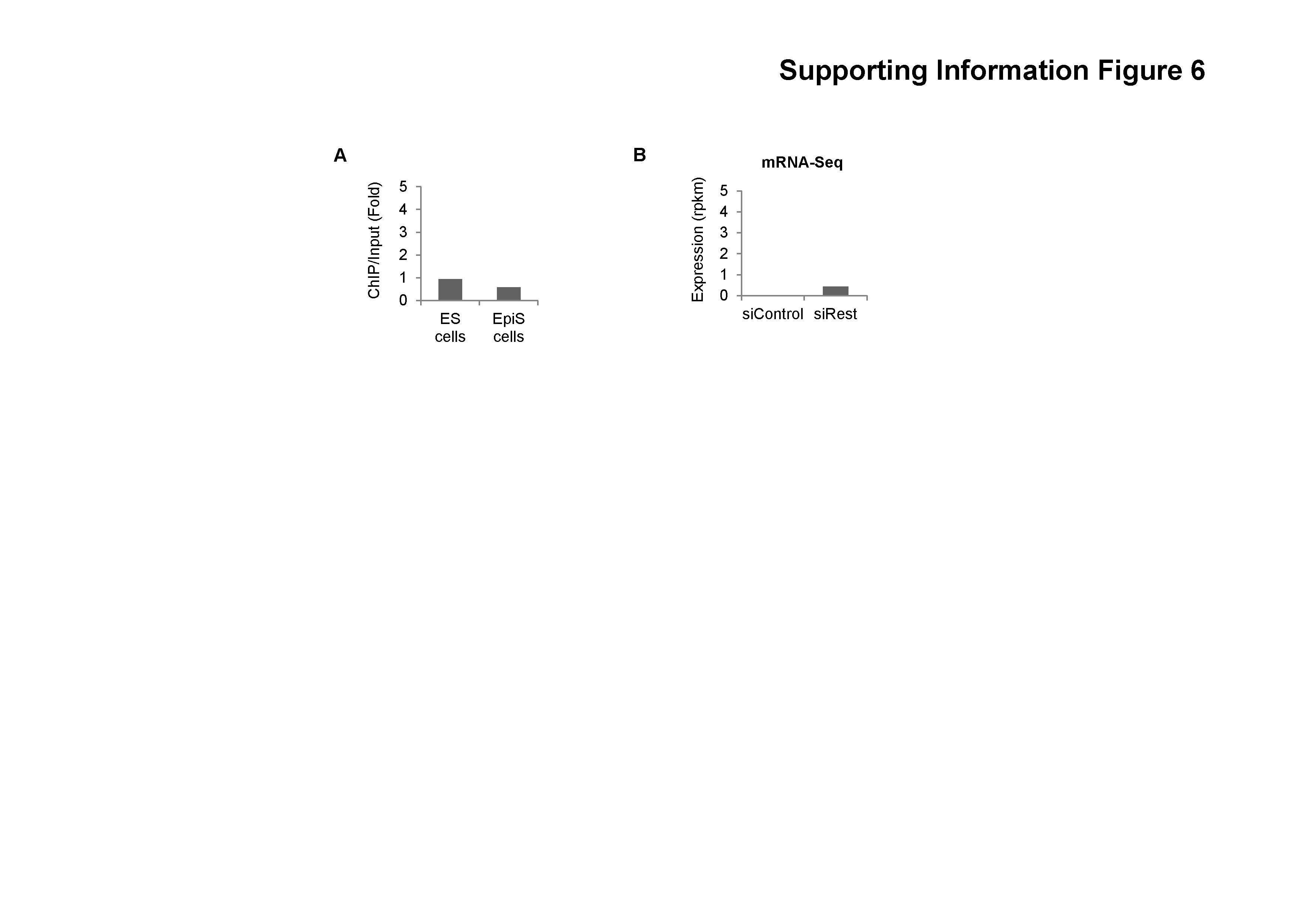

Supplement: Figure S6 — Validation of the effect of Rest to miR21. (A) Signal intensities of Rest binding on Rest binding site around miR21, indicated on [7], in ES and EpiS cells. (B) miR21 induction through Rest knock-down is shown. (TIFF) [file pone.0095374.s006.tif]
